# Th2-polarised PrP-specific Transgenic T-cells Confer Partial Protection against Murine Scrapie

**DOI:** 10.1371/journal.ppat.1002216

**Published:** 2011-09-01

**Authors:** Saci Iken, Véronique Bachy, Pauline Gourdain, Annick Lim, Sylvie Grégoire, Thomas Chaigneau, Pierre Aucouturier, Claude Carnaud

**Affiliations:** 1 UPMC Univ Paris 6, UMR_S 938, Centre de Recherche Hôpital Saint-Antoine, Paris, France; 2 INSERM, UMR_S 938, Centre de Recherche Hôpital Saint-Antoine, Paris, France; 3 Unité du Développement des Lymphocytes, Institut Pasteur, Paris and INSERM U668, Paris, France; University of Edinburgh, United Kingdom

## Abstract

Several hurdles must be overcome in order to achieve efficient and safe immunotherapy against conformational neurodegenerative diseases. In prion diseases, the main difficulty is that the prion protein is tolerated as a self protein, which prevents powerful immune responses. Passive antibody therapy is effective only during early, asymptomatic disease, well before diagnosis is made. If efficient immunotherapy of prion diseases is to be achieved, it is crucial to understand precisely how immune tolerance against the prion protein can be overcome and which effector pathways may delay disease progression. To this end, we generated a transgenic mouse that expresses the ß-chain of a T cell receptor recognizing a PrP epitope presented by the class II major histocompatibility complex. The fact that the constraint is applied to only one TCR chain allows adaptation of the other chain according to the presence or absence of tolerogenic PrP. We first show that transgene-bearing T cells, pairing with rearranged α-chains conferring anti-PrP specificity, are systematically eliminated during ontogeny in PrP+ mice, suggesting that precursors with good functional avidity are rare in a normal individual. Second, we show that transgene-bearing T cells with anti-PrP specificity are not suppressed when transferred into PrP+ recipients and proliferate more extensively in a prion-infected host. Finally, such T cells provide protection through a cell-mediated pathway involving IL-4 production. These findings support the idea that cell-mediated immunity in neurodegenerative conditions may not be necessarily detrimental and may even contribute, when properly controlled, to the resolution of pathological processes.

## Author Summary

It is generally accepted that prion-specific antibodies can protect against mouse scrapie infection. However, passive antibody therapy is limited to the lymphoinvasion stage of the disease. Active immunization has been attempted but the results have been disappointing. There is therefore a need for developing analytical models that will allow a fine dissection of the immune mechanisms at play in prion diseases and help distinguish between protective effects mediated by B cells and antibodies, and the effect of T cells. The aim of our study was to thoroughly examine T cell tolerance to the prion protein and to evaluate whether a pure specific population of T cells adoptively transferred to a normal host could proliferate and confer protection against scrapie. We designed a transgenic mouse in which the majority of T lymphocytes recognize the prion protein. Our key findings are that prion-specific T cells remain functional when transferred to normal recipients, even more so when the host is infected with scrapie, and confer partial protection against the disease by slowing down prion replication, in complete absence of anti-prion antibodies. Anti-prion T cells may therefore be considered as a therapeutic tool in the future.

## Introduction

Prion diseases, also termed transmissible spongiform encephalopathies (TSE), are fatal neurodegenerative disorders against which no treatment is available yet. The key pathogenic event is the conversion of the cellular prion protein (PrPc), a ubiquitous, host-encoded glycoprotein, into a misfolded protein, PrP scrapie (PrPSc) [1]. PrPSc forms oligomers that are self-propagating and cause neuronal damage. PrPSc is the presumed prion agent which is necessary and sufficient for disease transmission and gives strain-associated characteristics [2].

Many recent reports have shown that mice treated with antibodies (Abs) against PrP [3], [4], [5] or vaccinated against the protein [6], [7], [8] acquire resistance to scrapie peripheral infection. Encouraging as these findings may be, the results against TSE are not fully satisfactory yet and have precluded clinical trials. On one hand, passive Ab therapy is effective under restricted conditions, notably before neurological symptoms appear [4]. On the other hand, active vaccination is limited by the strong tolerogenicity of self PrP [9], [10]. With a few exceptions [6], vaccinated mice enjoyed only limited remission and ultimately succumbed to prion infection.

Focusing TSE immunotherapy on CD4^+^ T cells rather than on Ab-producing cells may overcome these difficulties [11]. Two strategies borrowed from cancer immunotherapy have already been probed for that purpose: dendritic cell (DC) vaccination [12] and adoptive CD4^+^ T cell therapy [13]. Improving T-cell-based immunotherapy, however, requires deeper insights into several issues. The way the anti-PrP repertoire is selected in the thymus and the chances that anti-PrP T cells with good functional avidity may overcome negative selection must be evaluated. One must also understand the way such lymphocytes, whether generated in the host or adoptively transferred, can be activated by prion-infected cells and neutralize prion progression. To address these issues, we have produced a transgenic (Tg) mouse expressing a single T cell receptor (TCR) β-chain from an anti-PrP TCR. The α-chains rearrange freely so that the anti-PrP repertoire adapts to the antigenic context. This model made it possible to follow repertoire development in PrP+ and PrP– mice through analysis of the α-chain rearrangements, and to produce highly enriched populations of T helper (Th) cells. The therapeutic efficiency of those T cells was assessed following adoptive transfer.

## Results

### Phenotypic and Functional Characterization of TCR-β Tg Mice on PrP+ or PrP–Backgrounds

**Figure 1:**
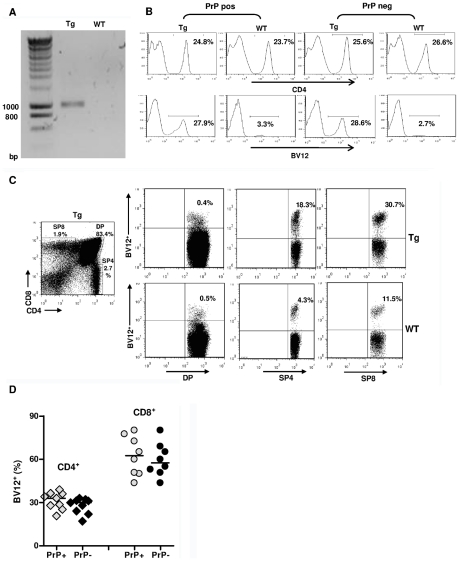
Expression of the TCR β-chain transgene in PrP+ and PrP– mice. (A) Genomic PCR from a founder mouse. (B) FACS analysis of LN cells from Tg versus WT mice bred on a PrP+ or PrP– background showing histograms of the percentages of BV12^+^ T cells in gated CD4^+^ populations. (C) Over-expression of the BV12^+^ transgene starting at the single positive stage of thymic differentiation. Tg mouse (top row) and WT mouse (bottom row). (D) BV12^+^ cells within CD4^+^ or CD8^+^ LN T cells of Tg PrP+ and Tg PrP– mice. Each symbol represents an individual mouse. Horizontal bars show medians. Differences within subsets are not significant.

Among three founders, only one B6 male (Figure 1A) over-expressed the transgenic beta variable (BV) 12^+^ rearrangement in peripheral blood cells (29.8% versus 2.5% in controls, data not shown), and transmitted this phenotype to its progeny. Spleens and lymph nodes (LN) of Tg mice had normal size and normal total white cell content. Spleens of PrP+ and PrP– Tg mice contained 90±5×10^6^ and 92±6×10^6^ cells versus 96±8×10^6^ cells for spleens of wild type (WT) littermates (n = 5, data not shown). Tg mice on both PrP+ and PrP– backgrounds displayed a minor reduction in the relative percentage of CD4^+^ T cells compared to CD8^+^ T and total B cells (Figure S1), suggesting that the expression of the TCR transgene had a slight impact on T cell ontogeny, as also reported in other TCR-Tg lines [14]. Secondary lymphoid organs from Tg mice showed fully developed germinal centers and the presence of cells with strong cell-surface PrPc expression (Figure S2A,B). The expression of PrPc was also similar in the brain between WT mice and Tg PrP+ mice, as assessed by immunohistochemistry and by FACS analysis (Figure S3A,B). Therefore, the transgenic insertion of the TCR β-chain in affects neither the architecture of secondary lymphoid organs nor PrPc expression.

As shown in Figure 1B, the BV12^+^ rearrangement was still dominant in the third generation of mice raised on PrP+ or PrP– backgrounds. The fact that endogenous rearrangements were not excluded and that only a third of total CD4^+^ T cells were BV12^+^ suggested a belated expression of the transgene due to the nature of the cassette. This inference was confirmed by flow cytometry analysis of thymocytes from Tg mice showing an increase in the percentage of transgenic BV12^+^ rearrangements only after they reach the single positive stage (Figure 1C). The expression of the β-chain transgene in the SP8 subset resulted from the deletion of the *Cd8* gene silencer, which is present in the original plasmid, but which happens to be excised during the construction process [15].

Figure 1D shows the respective percentages of transgene-bearing CD4^+^ and CD8^+^ LN lymphocytes in several PrP+ and PrP– Tg mice. Percentages of BV12^+^ T cells were practically identical in PrP– and PrP+ mice, and the higher percentage of transgene expression among CD8^+^ LN T cells confirms the known preferential affinity of BV12^+^ rearrangements for MHC class I products during thymic selection [16].

**Figure 2:**
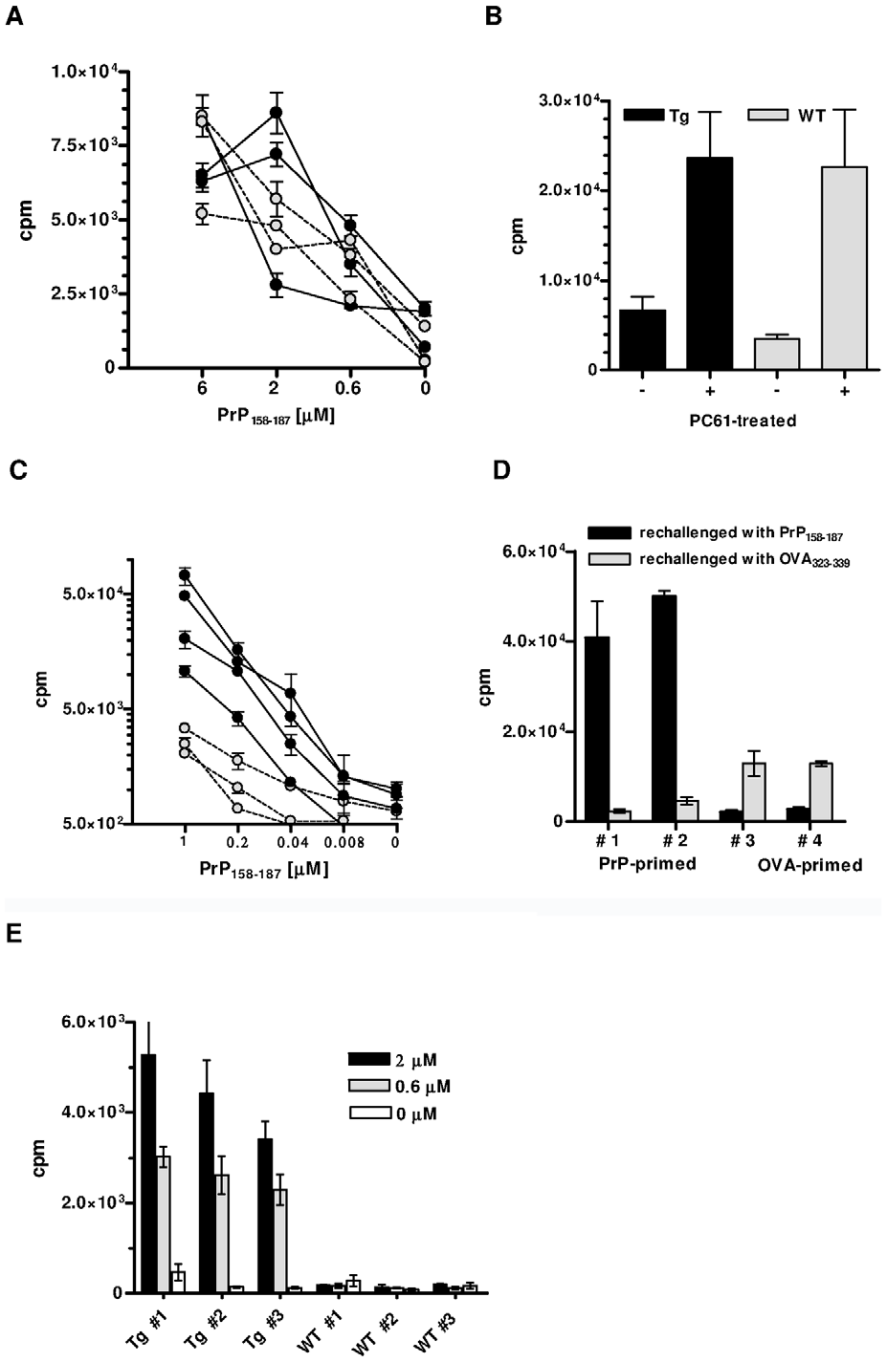
In vitro responses of CD4^+^ T cells from PrP+ or PrP– Tg mice. (A) Proliferation against PrP_158–187_ of PrP-primed CD4^+^ T cells from Tg (black circles) or WT (grey circles) PrP+ mice. T cells were collected 10 days after priming. Error bars show standard errors of triplicate cultures. Cultures were pulsed at day 5. (B) Proliferation of PrP-primed T cells from Tg or WT PrP+ mice treated with PC61 or an isotype control. T cells were challenged with 6 µM of PrP_158–187_. Cultures were arrested as in (A). (C) In vitro proliferation of PrP-primed CD4^+^ T cells from Tg (black circles) or WT (grey circles) PrP– mice. Cultures were pulsed at day 3. Error bars show standard error of triplicate cultures. (D) PrP-primed CD4^+^ T cells from Tg mice respond massively and specifically to peptide PrP_158–187_ but not to the irrelevant OVA_323-339_ peptide. Conversely, priming with OVA_323–339_ results in a moderate response to the peptide. (E) Primary responses to PrP_158–187_ of naive T cells from Tg or WT PrP– mice. Numbers in legend correspond to peptide concentrations present in the micro-cultures. Cultures were pulsed at day 5. Error bars show standard deviations of triplicate wells. Each graph is representative of at least 3 independent experiments, with 3 to 4 mice per group.

As reported [9], [10], PrP+ WT mice do not normally respond to PrP. Because PrP+ Tg mice expressed a high percentage of TCR β-chain rearrangements, we decided to examine whether, in contrast to WT mice, these mice would be responsive. As Figure 2A shows, in vivo primed T cells from Tg mice proliferated weakly and to the same extent as T cells from WT littermates. Therefore, either pairings with alpha rearrangements conferring anti-PrP reactivity were deleted, or PrP-responders escaping selection were suppressed, notably by regulatory T (Treg) cells [17]. To test the latter possibility, Tg PrP+ mice were in vivo deprived of Treg cells by anti-CD25^+^ T cell Ab (clone PC61) before being primed with PrP_158–187_ 4 days later. CD4^+^ T cells deprived of Treg lymphocytes proliferated more vigorously than untreated controls in response to antigenic challenge, but within the same range as non-Tg littermates similarly deprived of Treg cells (Figure 2B). Treg blockade therefore did not reveal the specific presence of PrP responders within the pool of transgene-bearing T cells.

The situation was remarkably different in PrP– Tg mice. In vivo primed CD4^+^ T cells from these mice proliferated almost five times as much upon in vitro challenge than non-Tg PrP– mice, which also respond to the peptide [18] (Figure 2C). T cells responded essentially to peptide PrP_158–187_ and only marginally to an I-A^b^-restricted peptide of ovalbumin (OVA_323–339_) (Figure 2D). Conversely, priming CD4^+^ T cells from Tg mice with OVA_323–339_ generated a modest, but specific response against ovalbumin which suggests that a few non-PrP precursors are present in the total CD4^+^ repertoire of Tg mice (Figure 2D). PrP_158–187_-specific IFN-γ-producing CD4^+^ T cells evaluated by ELISPOT were also substantially increased in Tg versus non-Tg mice (Figure S4). No IL-4 response could be detected under the same conditions, suggesting an initial shift of anti-PrP T cells toward a Th1 profile. A characteristic of biased T cell repertoires is their capacity to respond to primary in vitro stimulation [19]. Naive CD4^+^ T cells from Tg mice did indeed proliferate in response to PrP_158–187_ in a dose-dependent manner, in contrast to naive non-Tg T cells (Figure 2E). Expansion of the anti-PrP repertoire in PrP– Tg mice could also be evidenced by the fact that the percentage of BV12^+^ T cells in LN underwent a substantial increase after peptide priming (Figure S5).

### Analysis of the α-chain Rearrangements Pairing with the β-chain Transgene

**Figure 3:**
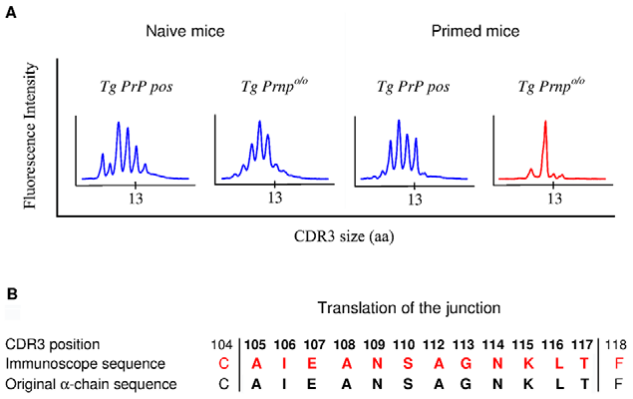
Immunoscope analysis of TCR α-chains pairing with the BV12^+^ TCR β-chain. (A) Focus on CDR3 diversity of TRAV13^+^ chains in naive or primed CD4^+^ cells from PrP+ and PrP– mice. (B) Identity between CDR3 sequences of TCR α-chains paired with the BV12^+^ TCR β-chain in PrP– mice and the CDR3 sequence of the original TCR α-chain identified in the T cell hybridoma. Each experiment is representative of 2 or 3 independent analyses, with a pool of at least 3 mice per test.

To gain insight into the mechanisms of repertoire selection on PrP+ and PrP– backgrounds, we analyzed the diversity of TCR α-chain rearrangements among sorted CD4^+^ BV12^+^ T cells by immunoscope. Figure S6 gives an overview of the 20 alpha variable chain (TRAV) families associated with the β-chain transgene in naive and primed Tg mice. Table 1 shows the actual percentages for each family. TRAV family usage did not differ between PrP+ or PrP– naive Tg mice. In particular, TRAV13, the variable segment constitutive of the α-chain of the original hybridoma that had donated the β-chain, was used to the same extent in the two Tg progenies. The pattern changed in primed BV12^+^ T cells. Whereas TRAV13 usage in PrP+ primed mice was practically unmodified, it represented almost 80% of all families in PrP– mice. Moreover, the length of the TRAV13 third complementarity determining region (CDR3) followed a Gaussian distribution except for the rearrangements in primed PrP– mice where the distribution presented a predominant peak of 12 residues (Figure 3A). This CDR3 was homogeneous enough to be sequenced directly from the PCR product. The sequence was identical to the α-chain CDR3 expressed in the original TCR hybridoma (Figure 3B).

**Table 1:** Relative percentages of TRAV family usage within the TCR α-chains pairing with the transgenic TCR β-chain.

	Tg naive		Tg primed	
	PrP–	PrP+	PrP–	PrP+
AV1	0.0	0.0	0.0	*0.4*
AV2	0.0	0.0	0.0	*0.2*
AV3	0.9	0.7	0.0	*4.2*
AV4	2.4	31.7	10.5	*6.2*
AV5	5.1	0.0	0.0	*0.9*
AV6A	2.0	0.6	0.0	*1.8*
AV6B	2.7	0.7	0.0	*1.2*
AV7	11.5	14.9	1.1	*27.7*
AV8	4.0	4.0	0.0	*3.2*
AV9	24.7	7.2	3.2	*16.0*
AV10	1.8	0.1	0.0	*0.8*
AV11	7.5	0.1	0.0	*1.1*
AV12	3.8	9.8	4.7	*9.8*
AV13	25.2	25.9	**79.8**	*15.6*
AV14	3.3	3.9	0.7	*7.5*
AV15	0.0	0.0	0.0	*0.5*
AV16	0.0	0.1	0.0	*0.6*
AV17	2.5	0.8	0.0	*1.3*
AV18	0.0	0.0	0.0	*0.0*
AV19	0.0	0.0	0.0	*0.4*
AV21	2.6	0.3	0.0	*0.5*

### Fate of Transgene-bearing CD4^+^ T Cells Transferred into Normal or Infected Recipients

**Figure 4:**
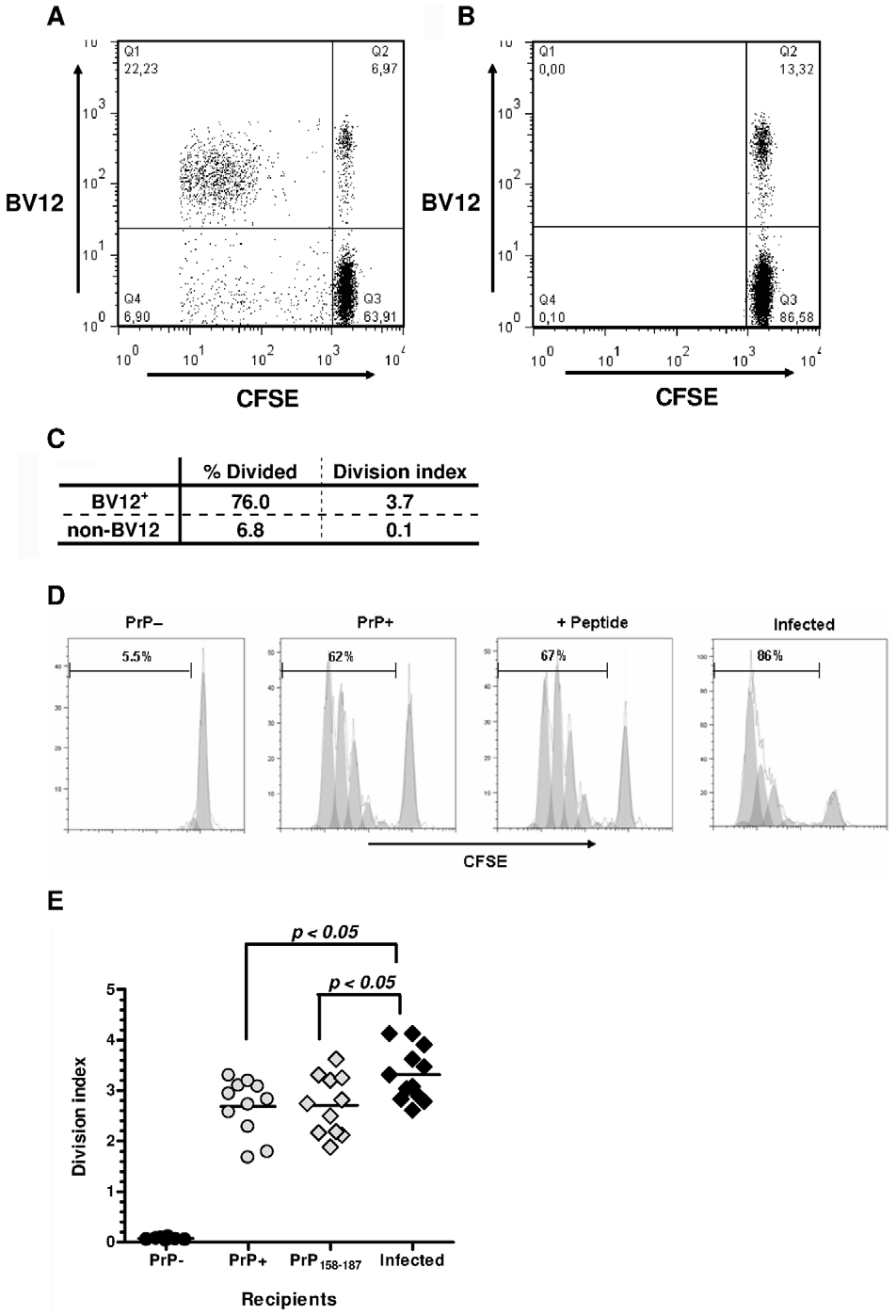
In vivo proliferation of CD4^+^ BV12^+^ cells. (A) Dot-plot of CFSE-labeled CD4^+^ T cells after 3-day engraftment in mice boosted with PrP_158–187_. Cells were gated in a Ly5.2^+^ window. (B) Same cells as in (A) but transferred into non-loaded PrP– mice. (C) Proliferation statistics on CD4^+^ BV12^+^ and BV12^-^ T cells displayed in (A). (D) Modelized representation of T cell divisions in various recipients. Digits in each histogram represent the “% Divide”. (E) Division indexes of CD4^+^ BV12^+^ cells transferred into various recipients: PrP– (n = 8), PrP+ (n = 11), peptide-loaded PrP– (n = 11), prion-infected (n = 12). Each symbol represents a single mouse. Horizontal bars show the means. Differences between the infected mice and the other groups were significant by one-way analysis of variance (p<0.005) and by Bonferroni's multiple comparison tests.

Primed Ly5.2^+^ CD4^+^ T cells from Tg PrP– donors were labeled with carboxyfluorescein diacetate succinimidyl ester (CFSE) and injected into Ly5.1^+^ PrP+ and PrP– recipients. BV12^+^ lymphocytes among Ly5.2^+^ CD4^+^ gated T cells collected after a 3-day engraftment into PrP+ hosts had markedly proliferated, as shown by the presence of a resolved population in the upper left quadrant (Figure 4A). Far fewer non-BV12 CD4+ T cells were seen in the lower left quadrant. As expected, T cells injected into PrP– mice did not respond (Figure 4B). The “% divided” measuring the number of lymphocytes that had undergone mitosis and the average number of divisions per cell (division index) was much higher in the BV12^+^ than in the non-BV12 subset, indicating that responders were considerably more numerous and still reactive in the PrP+ environment (Figure 4C).

Next, we compared the proliferation of BV12^+^ CD4^+^ T cells in prion-infected versus healthy mice (Figure 4D). Of interest, the percentage of dividing T cells was strikingly higher in prion-infected mice. The same trend can be observed in Figure 4E, recapitulating data of more than 10 mice per group. Differences were statistically significant between infected and normal recipient mice (*p*<0.05 by ANOVA and Bonferroni's two-by-two comparisons). Infected antigen presenting cells (APCs) reactivated anti-PrP T cells more efficiently than non-infected APCs.

### Adoptive Transfer of Limited Amounts of Transgene-bearing CD4^+^ T Cells Delays Scrapie Onset in Peripherally Infected Mice

**Figure 5:**
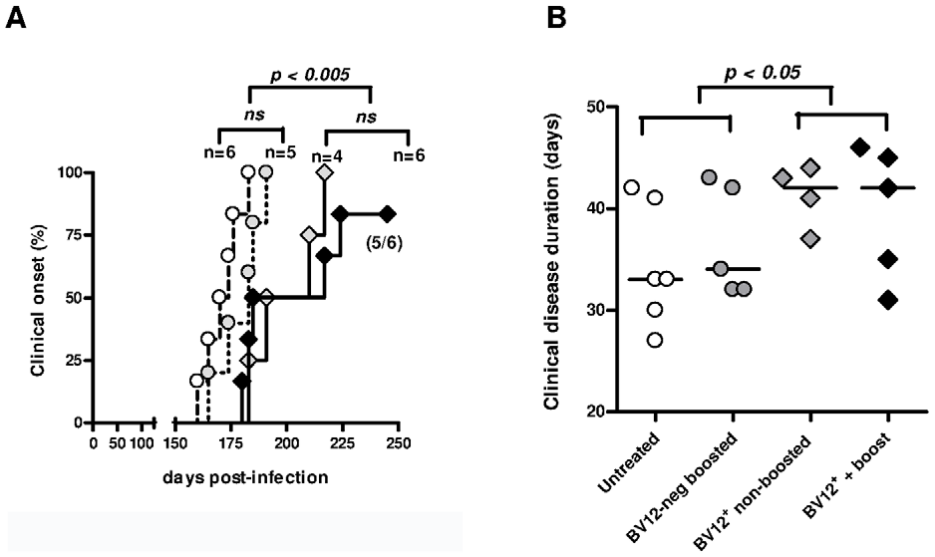
Adoptive transfer of minimal amounts of transgene-bearing CD4^+^ T cells delays scrapie onset and prolongs the clinical phase. (A) Disease onset in untreated controls (white circles, dotted line), in recipients of BV12-negative peptide-boosted T cells (grey circles, dotted line), in non-boosted recipients of BV12^+^ cells (grey diamonds, solid line), and in boosted recipients of BV12^+^ cells (black diamonds, solid line). Differences were significant between the 4 groups by multivariate log rank test (*p* = 0.0073). They were also significant (*p*<0.005) between the two control groups and the boosted plus non-boosted recipients of BV12^+^ T cells. (B) The clinical phase of scrapie was significantly longer in mice receiving BV12^+^ CD4^+^ T cells, whether boosted or non-boosted, compared with mice receiving BV12-negative CD4^+^ T cells or left untreated (p<0.05 by Mann-Whitney test between the two groups receiving BV12^+^ T cells and the two groups receiving BV12-negative T cells or no T cells).

Having previously shown that PrP-sensitized polyclonal T cells attenuate scrapie evolution [13], we undertook to confirm those conclusions with transgene-bearing CD4^+^ T cells. Two ×10^5^ CD4^+^ BV12^+^ primed T cells were transferred after sorting into CD3ε^o/o^ mice which had been infected 1 day before with 2×10^4^ LD_50_ of the prion strain 139A. Recipients of BV12^+^ T cells were boosted with peptide PrP_158–187_ one day after transfer and once monthly thereafter or left non-boosted. Controls consisted of mice transferred with sorted BV12-negative CD4^+^ T cells further boosted and of non-transferred mice. Neurological symptoms appeared in two waves. Non-transferred mice and mice transferred with BV12-negative T cells became sick at 170 and 178.5 median day post-infection (dpi), respectively, whereas recipients of BV12^+^ T cells either boosted or not became sick at 217 and 197 median dpi, respectively (Figure 5A). One out of the 6 BV12^+^ T cell transferred and boosted mice remained free of symptoms (Figure 5A). Differences in kinetics were statistically significant among the four groups according to multivariate log rank test, as well as between the two control groups compared to the two experimental groups which had received BV12^+^ T cells, but not between boosted versus non-boosted recipients of transgene-bearing T cells nor between the two control groups which received BV12-negative T cells or no T cells. Overt disease duration from onset to terminal stage was significantly longer in the two experimental groups treated with BV12^+^ T cells than in the two control groups (42 median day versus 33 median day respectively) (Figure 5B). Brains of mice culled at terminal stage contained similar amounts of proteinase-K (PK) -resistant PrP, irrespective of the nature of the T cell transfer, the clinical onset or the overt disease duration (Figure S7A). This suggests that terminal stage occurs when a sufficient amount of PK-resistant PrP has accumulated in the brain. In contrast, PK-resistant PrP could not be detected in the spleen and brain of the mouse which had remained permanently free of neurological symptoms and was sacrificed at 350 dpi (Figure S7B).

**Figure 6:**
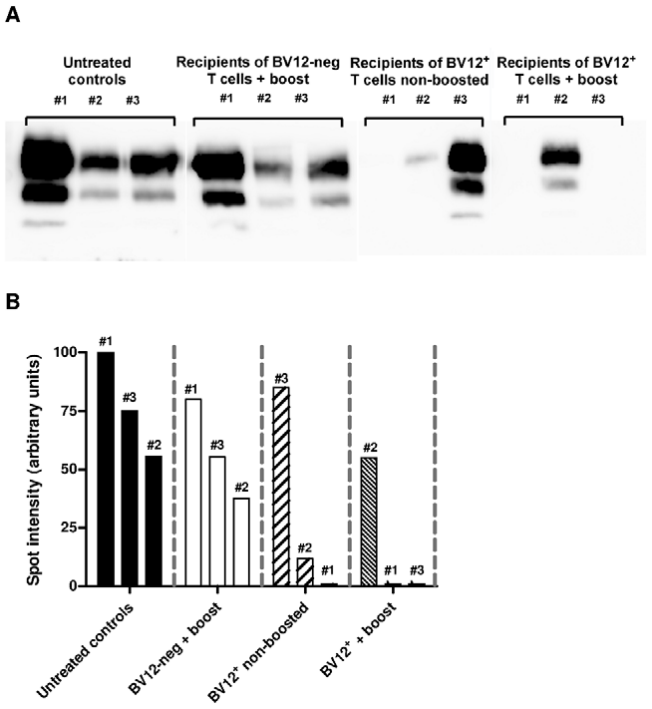
Anti-PrP T cells prevent the accumulation of PK-resistant PrP in secondary lymphoid organs. (A) Western blots of spleen material at 90 dpi. (B) Normalization of western blots shown in (A). Numbers above bars correspond to lanes in (A).

**Figure 7:**
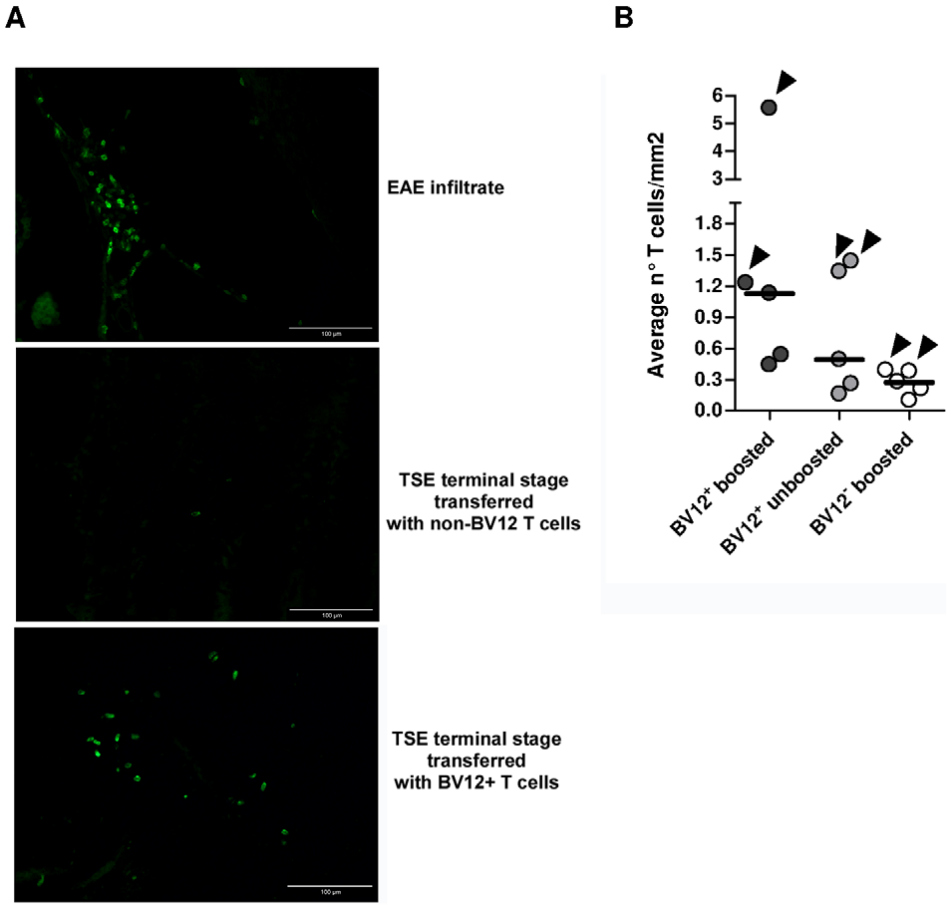
Presence of infiltrating T cells in the brain of adoptively transferred mice. (A) Frozen sections of anterior, medium, and posterior crosswise brain portions showing the presence lymphocytes staining positively with an anti-CD3 Ab. The EAE brain culled at the climax of MOG-induced disease served as a positive control (40X). (B) Summary of infiltrating T cell frequencies in brains of adoptively transferred mice. Each circle represents the frequencies of infiltrating T cell of an individual mouse, based on the analysis of at least 12 sections from anterior, median and posterior brain portions. Arrows indicate infiltration values at terminal stage while the other dots show infiltration values at 90 dpi.

To get an insight on where T cells exert their prionostatic effects, two types of experiments were performed. First, a few experimental and control mice were sacrificed at 90 dpi in order to compare the levels of PK-resistant PrP in their spleens by western blot. Second, we looked for the presence of T cells in the brain of the above mentioned mice sacrificed at 90dpi and of mice sacrificed at terminal stage. As shown in Figure 6, 4 out of 6 spleen samples of mice that had received BV12^+^ CD4^+^ T cells (either boosted or non-boosted), were almost completely free of pathological PrP, suggesting that anti-PrP T cells blocked the propagation of PK-resistant PrP in peripheral lymphoid tissues. At terminal stage, T cells were present in the brains of all mice which had received BV12^+^ T cells, whether boosted or non-boosted. At 90 dpi, one mouse transferred with BV12^+^ T cells and boosted already showed CNS infiltration. Fewer infiltrating T cells were detected, even at terminal stage in the brains of mice that had received BV12-negative T cells (Figure 7).

### Humoral and Cell-mediated Anti-PrP Responses in Adoptively Transferred Mice

Blood samples were collected at 90 dpi to assess the presence of donor T cells in the CD3ε^0/0^ hosts (Figure S8). CD4^+^ T cells were clearly evidenced among the peripheral blood lymphocytes of transferred mice. The expansion of BV12^+^ CD4^+^ T cells was virtually identical in mice which had been boosted with peptide PrP_158–187_ or in non-challenged mice.

**Figure 8:**
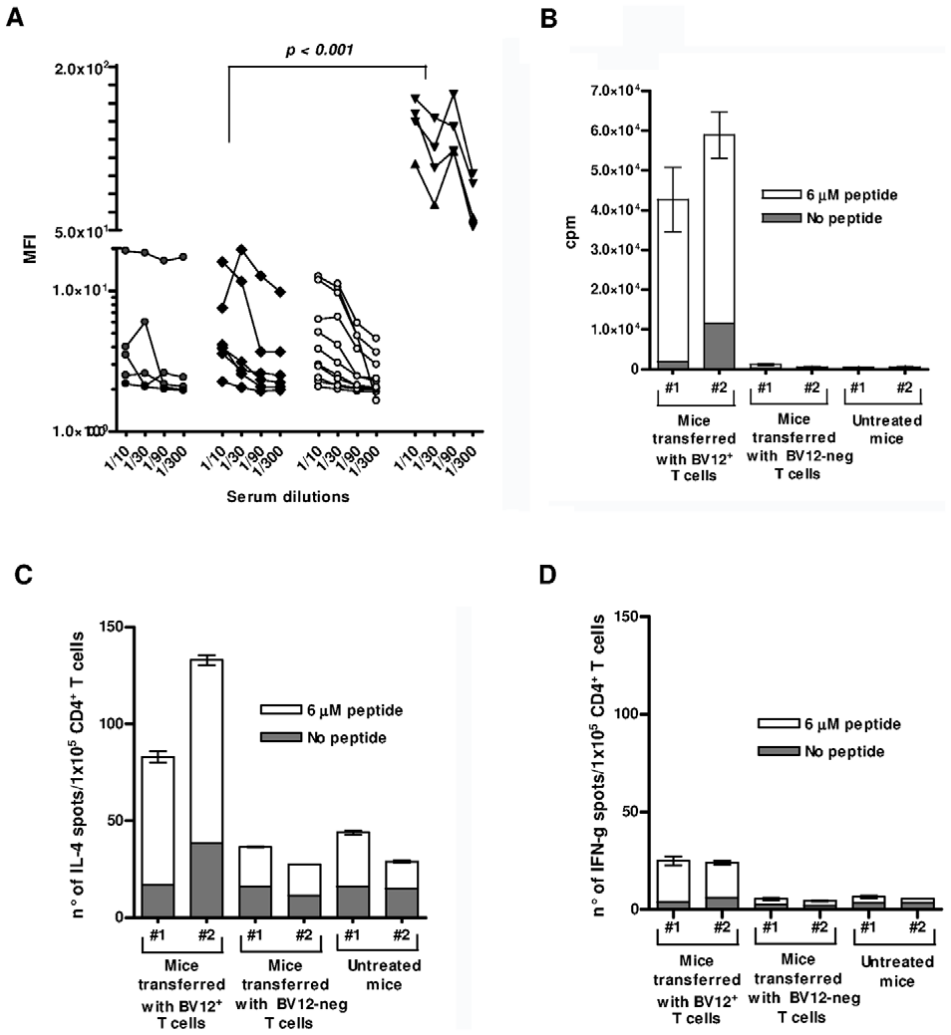
Humoral and cell-mediated responses of adoptively transferred T cells. (A) Anti-PrP Abs against cell-surface PrPc. Sera were collected at 90 and 120 dpi. They are presented collectively since the time of collection had no impact on MFI values. Cell-bound Abs was revealed by immunofluorescence. Black circles represent sera from non-transferred mice, black diamonds sera from mice transferred with BV12-negative T cells, gray circles sera from mice transferred with BV12^+^ T cells boosted and non-boosted, black triangles are positive controls from PrP– Tg mice primed with peptide PrP_158–187_. MFIs (at the 1∶100 dilution) between positive controls and the 3 other groups were significantly different by the Kruskal-Wallis test. (B) In vitro proliferation of spleen T cells collected at 120 dpi from recipient mice. Error bars show the standard deviations of triplicates. Each column represents an individual mouse. (C) ELISPOT assay of IL-4-secreting T cells collected at 120 dpi. (D) Same as in (C) for IFN-γ secretors.

Serum samples were collected at 90 and 120 dpi to monitor for the presence of circulating Abs against native PrPc which presumably convey protection (Figure 8A). Abs were barely detectable in all serum samples, regardless of collection time and of treatment. Transferred T cells therefore had not initiated a productive T-B cooperation with host B lymphocytes. Next, we looked at antigen-specific T-cell proliferation and lymphokine secretion at 120 dpi. Engrafted BV12^+^ T cells could still proliferate in response to PrP_158–187_ (Figure 8B). A substantial proportion of T cells also released IL-4 after antigenic challenge, as evidenced by ELISPOT (Figure 8C), but at variance with freshly activated anti-PrP T cells, long-term engrafted lymphocytes were low producers of IFN-γ (Figure 8D). Contrasting with those low values, control T cells stimulated with concanavalin A displayed a frequency of IFN-γ secretors in the range of 200 spots per 1×10^5^ cells (data not shown).

## Discussion

The mechanisms at work in prion immunotherapy are still poorly understood. Abs against PrP or PrP receptors have been considered to be the major protagonists so far, following the demonstration that they cured infected cell lines in vitro [20], [21] and retarded disease progression in vivo [3], [4], [5], [22]. By contrast, many authors have seen the involvement of T cells in neurodegenerative conditions as counterproductive, based on the fact that T cells may promote neuronal decay via microglia [23] or initiate autoimmune complications such as reported in Alzheimer's disease patients [24]. Recent evidence suggests, however, that in addition to providing help to B cells, CD4^+^ T cells may also make a contribution of their own against neurodegenerative conditions [25].

Our first objective was to find out whether anti-PrP T cells with good TCR functional avidity may escape central tolerance and be available for active vaccination or adoptive therapy. With the use of single β-chain Tg mice we were able to show how anti-PrP precursors emerge in a PrP+ or negative environment. A first conclusion is that the preselected PrP repertoire in the thymus is tightly controlled, leaving little chance for lymphocytes with good TCR avidity to escape selection and to settle in peripheral organs. Although the percentages of transgene-bearing CD4^+^ T cells in PrP+ Tg mice are as high as in PrP– mice, their reactivity to PrP peptide is as limited as that of T lymphocytes from non-Tg littermates. The blockade of CD25^+^ regulatory T cells prior to antigenic challenge increased proliferation and IFN-γ release to the same extent as it did for T cells from non-Tg mice. Therefore, Treg cells cannot presently account for the lack of responsiveness of PrP+ Tg mice even though a regulation of anti-PrP precursors by foxp3^+^ T cells has been described [7]. The situation is totally different in PrP– Tg mice in which CD4^+^ T cells displayed strong reactivity against the dominant PrP epitope. They proliferated more vigorously upon antigenic challenge than non-Tg littermates, producing more IFN-γ and responding in vitro even without in vivo priming. Although the TCR α/β repertoire against a given antigenic specificity is generally considered to be highly diverse, limited heterogeneity of β-chain rearrangements has been reported [26], [27], [28]. Limited heterogeneity is also reflected by the fact that the T cell repertoire of single TCR β-chain Tg mice is frequently skewed toward the specificity of the TCR that provided the rearranged chain [19]. But, as shown here, the β-chain is not sufficient by itself to impart TCR specificity since BV12^+^ T cells in PrP+ mice were unresponsive to PrP. As other studies have demonstrated, the α-chain makes an essential contribution to the final specificity and determines ultimately whether a T cell precursor responds or not to a defined epitope [28], [29], [30], [31]. Central selection in PrP+ mice probably eliminates the alpha pairings that confer anti-PrP responsiveness. This phenomenon is all or none because α pairings that would result in TCRs of intermediate avidity are visibly not spared either. Selection based on the choice of a TCR α-chain rearrangement is one of many strategies aimed at preventing autoreactivity [32]. A different TCR β-chain transgene originating from a TCR with lower functional avidity would possibly have allowed pairing with α-chains conferring responsiveness, as evidenced by the generation of activated T cells in normal WT mice challenged with immunogenic formulations of PrP.

Immunoscope analyses confirmed those conclusions. No bias in TRAV family usage was identified in naive CD4^+^ BV12^+^ T cells in a PrP+ or -negative context. This indicates a wide variety of α pairings among exiting T cells even though anti-PrP precursors are already abundant in the naive repertoire of PrP– Tg mice, as shown by primary in vitro responses. However, after antigen selection, the distribution of TRAV families differed radically in PrP+ and PrP– mice. TRAV13 usage in primed PrP+ Tg mice was similar to that of naive mice, whereas TRAV13 usage became predominant in primed T cells from PrP– Tg mice. Similar changes in α-chain diversity after antigenic priming were demonstrated in single-chain Tg mice challenged with lymphocytic choriomeningitis virus (LCMV) and in Tg non-obese diabetic (NOD) mice spontaneously developing autoreactive T cells against islets of Langerhans [29], [30]. Noteworthy, this dominating TRAV13 rearrangement showed a highly homogeneous CDR3 domain. The CDR3 nucleotide sequence was identical to that of the α-chain isolated in the original T cell hybridoma. From a practical point of view, this result shows that after antigen priming, the BV12^+^ T cell subset is composed of a quasi-monoclonal population of anti-PrP effector cells. A similar result was described in a single-chain Tg NOD mouse, in which CD8^+^ T cells had infiltrated the pancreas and had been activated locally [30].

Another valuable conclusion, from the perspective of adoptive T cell therapy, is that anti-PrP T cells maintain their reactivity in a PrP+ environment and are preferentially activated by prion-infected cells. CFSE-labeled CD4^+^ T cells from primed Tg mice showed vigorous proliferation after a 3-day engraftment in PrP+ mice or in PrP– mice immunized with peptide. As expected, they did not proliferate in non-challenged PrP– hosts, confirming that the response was antigen specific and not caused by homeostatic regulation [33]. Contrasting with the highly rigid selection at work in the thymus, the periphery appears to be more permissive, notably to primed T cells. As previously suggested [13], naive anti-PrP precursors may be more receptive to peripheral regulation. It is worth noting that, far from being inhibited, specific T cells proliferated more vigorously in scrapie-infected mice. The quantitative parameters indicated that the increased proliferation results from more BV12^+^ T cells being activated and undergoing division than from an increased number of divisions within the 3 days of engraftment. Several reasons could account for the observed heightened T cell proliferation. Prion-infected APCs might present a higher density of MHC class II molecules filled with PrP peptide (signal 1), thus activating T cells more efficiently. A non-mutually exclusive alternative could be that prion-infected APCs provide more co-stimulation (signal 2) to T cells than healthy APCs. Preliminary examination of CD80 and CD86 expression on DCs collected from infected mice did not favor this latter hypothesis (data not shown), but other co-stimulatory pathways deserve to be examined. Finally, because PrPc is mobilized at the immunological synapse [34], the possibility that transconformed PrPSc might enhance APC function cannot be totally ruled out, even though this pathway remains difficult to conceive given the structural properties of the conformer.

In accordance with previous observations [13], sensitized anti-PrP T cells passively administered into infected mice convey protection with no evidence of negative side effects. Presently, as few as 2×10^5^ primed BV12^+^ T cells delay neurological onset and overt disease progression to the same extent as 50 times more polyclonal T cells [13]. The necessity of boosting Tg T cells seems less imperative than with polyclonal T cells, a fact which is consistent with the observation that PrP-specific T cells can be re-activated in vivo by prion infected-APCs or by APCs naturally processing endogenous PrPc, with no need to boost with exogenous peptide.

The lack of detectable anti-PrP Ab in T-cell transferred mice argues against the recruitment of resident B cells and the involvement of Abs in delaying disease progression. As previously reported [9], anti-PrP B cells differentiating in a PrP+ context, which is the situation in the recipient, are severely repressed and few precursors remain available in the periphery. In addition, by sorting donor cells for maximum purity, we made sure that donor B cells were not co-transferred. A T-cell-mediated pathway therefore seems to be the most likely explanation for the observed disease attenuation. T cells activated ex vivo after a 120-day engraftment still proliferate in the presence PrP peptide and produce substantial amounts of IL-4. We previously reported that a hallmark of polyclonal CD4^+^ T cells conveying protection was the production of both IFN-γ and IL-4 [13]. In the present experimental setting in which small numbers of highly enriched effectors were transferred, we can further suggest that protection is conferred by anti-PrP T cells that produce IL-4 rather than IFN-γ. Anti-PrP T cells, which are predominantly biased toward IFN-γ release shortly after priming, seem to evolve with time toward a Th2 oriented profile. In future experiments, the transfer of homogeneously polarized lymphocytes should confirm whether a Th2 profile preferentially conveys protection.

Where and how do T cells precisely halt disease progression? A reasonable hypothesis, also supported by the reduced content of PrPSc found in spleens of protected mice, is that PrP-sensitized T cells exert their prionostatic effects in the periphery, when prions propagate into secondary lymphoid tissues. The delayed clinical onset and protracted disease evolution would thus be a direct consequence of the slowed down lymphoinvasion, as already demonstrated in situations where the connection between follicular dendritic cells and nervous endings is perturbed [35] or when DCs or follicular dendritic cells are temporarily deleted [36], [37], [38]. Anti-PrP T cells would preferentially be activated at the sites of prion expansion such as in germinal centers, upon contact with infected DCs [39]. They would shift progressively toward a Th2 profile, and would mobilize agents of innate immunity such as alternatively-activated macrophages with a capacity to reduce inflammation and to degrade infectious oligomers in lymphoid follicles [40], [41], thereby retarding lymphoinvasion. But extraneural and neural invasion are not necessarily connected and the two processes could evolve independently [42], [43]. It is then conceivable that protective T cells exert their prionostatic effects in parallel, in peripheral lymphoid tissues and in the CNS. Both the extension of clinical disease duration and the detection of infiltrating BV12^+^ T cells in the brain of adoptively transferred mice speak in favor of a central action of T cells. Beneficial effects of IL-4-producing T cells have been described in various CNS pathological conditions as well as in physiological situations of memory acquisition [44], [45], [46]. Microglial cells or blood borne macrophages alternatively activated by Th2 T cells might retain their innate capacity to degrade pathological oligomers or fibrils [47] while protecting neurons from PrPSc-mediated toxicity. A recent study showing that prion propagation and neurotoxicity are dissociated processes [48] further supports the idea that the protective role of alternatively-activated effectors of innate immunity may not be confined to phagocytosis of amyloid aggregates only. Microglia activated by IL-4 induces oligodendrogenesis from adult stem cells. This effect is mediated, at least in part, by insulin-like growth factor-1 [49]. IL-4 can also counteract, in a dose-protective manner, the harmful effect of microglia activated by LPS. The mechanism by which IL-4 exerts its neuroprotective effects, involves the decrease of TNF-α and nitric oxide production [50]. The role of IL-4 in regulating CNS inflammation was also investigated in experimental autoimmune encephalomyelitis, a mouse model for multiple sclerosis. Ponomarev et al. showed that mice deficient in IL-4 had exacerbated neurological symptoms associated with a significant increase in infiltrating inflammatory cell number. CNS-resident microglial cells expressed in an IL-4 dependent manner the protein Ym1, a marker of alternatively activated macrophages [51].

In conclusion, our present results provide further support in favor of a positive role of cell-mediated immunity in TSE. T cells are not necessarily detrimental. They can be useful depending on their differentiation profile and, most likely too, on the stage of disease evolution. More generally, the success of immunotherapeutic strategies against neurodegenerative conditions will depend on our capacity to draw a clear line between useful and harmful processes and to set up beneficial synergies between the different arms of innate and acquired immunity.

## Materials and Methods

### Ethic Statement

All animal procedures were carried out in strict accordance with the French legislation (Rural Code articles L 214-1 to L 214-122 and associated penal consequences) and European Treaty ETS 123 (1986). They were approved by the Regional Ethic Committee “Charles Darwin” to which the animal facility is affiliated.

### Mice

PrP– mice (*Prnp^0/0^*) [52] were propagated on a C57BL/6 (B6) background [13]. Ly5.1 and CD3ε^0/0^ mice were also on a B6 background.

### Generation of TCR-β Transgenic Mice

A rearranged TCR β-chain (TRBV12-1-01*/TRBD1-01*/TRBJ1-4-02* (http://imgt.cines.fr)) was cloned from a T hybridoma generated by fusion of T cells from PrP– mice immunized with peptide PrP_158–187_ [18]. Genomic DNA was inserted into an expression cassette containing the *Cd4* promoter [15]. The *Cd8* silencer, originally present, was ultimately excised from the construct. The backbone vector was a PNNO3 plasmid, and the transgene was inserted at a *Sal*I restriction site and excised with *Not*I. Purified DNA fragment was microinjected into WT B6 eggs (SEAT facility, Villejuif, France). Founders were identified by tail PCR, and the selected founder was mated with PrP+ and PrP– breeders to establish two independent lines. The transgene was kept heterozygous in both progenies.

### Flow Cytometry Analyses

Transgene expression was followed with anti-Vβ5.1/5.2 Ab (clone MR9-4) conjugated to FITC (BD Biosciences, Pont-de-Claix, France). Other reagents were CD4-PE, CD8-PerCP-Cy5.5, CD19-PE, and Vβ5.1/5.2-biotinylated revealed by streptavidin-APC, all from BD Biosciences. Samples were analyzed with BD CellQuest or FlowJo (TreeStar, Ashland, Oregon, USA).

### In Vivo Priming and Enrichment of CD4^+^ T Cells

Mice received 50 µg of peptide PrP_158–187_ in complete Freund's adjuvant. Spleens and LNs were collected 10 days later. CD4^+^ T cells were enriched above 90% purity using a negative isolation kit according to the manufacturer's recommendations (Dynal, Invitrogen, Oslo, Norway).

### Functional in Vitro Assays

CD4^+^ T cells were assayed for proliferation in micro-culture plates containing mitomycin-C-treated PrP– spleen cells and various concentrations of PrP_158–187_ as previously described [18]. Cultures were pulsed overnight with tritiated thymidine after a variable number of days (indicated in figure legends). Lymphokine-secreting T cells were enumerated by ELISPOT assay according to standard procedures [34].

### Follow-up of CD4^+^ T Cell Activation in Vivo by CFSE Staining

Enriched CD4^+^ T cells (1×10^7^ cells) were labeled with 2.5 µM of CFSE (Molecular Probes, Invitrogen, Cergy-Pontoise, France) for 15 minutes in PBS at 37°C, washed twice with PBS-FCS 3%, and resuspended in PBS. Cells were injected i.v. at 2×10^6^ per mouse. Specific proliferation was measured at day 3 on a LSR2 cytometer (BD). Cells were also stained with Vβ5-biotinylated-streptavidin−PerCP-Cy5.5, CD4-APC-Cy5.5 and CD45.2-APC. Results are expressed as “% divide” (% of cells which underwent divisions) or “division index” (the average number of divisions that a cell undergoes, including cells that did not divide).

### Immunoscope Analysis of TCR α-Chain Repertoires

The diversity of α rearrangements was analyzed by immunoscope [53]. TRBV12^+^ T cells were sorted on a cell sorter (FACS-ARIA BD). Total RNA was prepared using a micro kit (RNeasy, QIAGEN, Courtaboeuf, France), and cDNA was synthesized with SuperScript II Reverse Transcriptase (Invitrogen). The distribution of TCR-Vα germline genes clustered into 20 families (IMGT nomenclature) was obtained by PCR. The relative usage of each Vα family was calculated according to the formula:
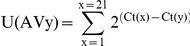
in which Ct(x) is the fluorescent threshold cycle number measured for a Vα (y) family.

For immunoscope profiles, products were subjected to run-off reactions for three cycles with a nested fluorescent primer specific for the constant region. The fluorescent products were separated and analyzed using an ABI-PRISM 3730 DNA analyzer. The size and intensity of each band were analyzed with “Immunoscope software” [53] further adapted to the capillary sequencer [54]. Fluorescence intensities were plotted in arbitrary units on the y axis, and CDR3 lengths (in amino acids) on the x axis.

### Monitoring of Scrapie Infection

Mice of both sexes, between 8 to 12 weeks of age, were infected i.p. with a brain homogenate of 139A prions containing 2×10^4^ LD_50_. Mice were monitored twice a week for gait control on a set of parallel bars [39].

### Detection of PK-resistant PrP by Western Blot

Spleens and brains collected at various time points post-infection were homogenized with the Ribolyser method and adjusted at 10% w/v in PBS plus a cocktail of anti-proteases. Precipitation with sodium phosphotungstic acid (Sigma) was performed as previously described [12]. Aliquots equivalent to 6 mg were PK-digested or left undigested. PK (proteinase K, Roche) was applied at 50 µg/ml, at 37°C for 30 min. Samples were run on 12.5% SDS-PAGE. PrP was revealed with Ab SAF-84 at 1/5000 (generously provided by Dr. J. Grassi, CEA). Signal was captured with a Fujifilm LAS3000 camera and quantified with a Fujifilm software program.

### Serological Detection of Abs against Native PrPc

Abs against native membrane-bound PrPc were measured by indirect immunofluorescence [18]. Sera were assayed at three-fold consecutive dilutions. Results are shown as geometric mean fluorescence intensities (MFIs).

### Histology

Hematoxylin eosin staining was performed on paraffin embedded sections at 5 µm, according to standard procedures. PrPc staining was performed on frozen sections of spleens and brains. Endogenous peroxydases were saturated with a solution of PBS-H_2_O_2_ at 0.3% final (Sigma, France) for 30 minutes. PrPc was labelled with a biotinilated SAF-83 Ab (gift of Dr. J. Grassi) at 5 µg/ml final concentration in tween-PBS for 1 hour at room temperature. Revelation was made with a horseradish-peroxidase streptavidin conjugate (GE Healthcare, Pittsburgh, USA) followed by incubation with a solution of diaminobenzidine (Diagnostics Biosystems, Pleasantville, USA).

Infiltrating T lymphocytes were identified on frozen crosswise brain sections as previously described [13]. Ten µm thick sections were air dried, fixed in acetone at 4°C and stained with an anti-CD3 rabbit monoclonal Ab (clone SP7) (Thermo Fisher Scientific, Fremont, USA) at 5 µg/ml. Revelation was achieved with a goat anti rabbit alexa fluor 488 (Invitrogen, Cergy-Pontoise, France).

Microphotographs were taken with an Olympus BX61 microscope equipped with an Olympus DP71 camera. Lens magnification is given in the legends to figures. Section areas were calculated with imagej software (http://rsbweb.nih.gov/ij).

### Statistical Analysis

Analyses were performed with GraphPad software (San Diego, CA, USA).

## Supporting Information

Figure S1 **Percentages of CD4^+^, CD8^+^, and B cells in Tg mice.** Percentages were measured by flow cytometry. CD4^+^ T cell proportion in Tg mice is slightly but significantly reduced (p<0.001 by ANOVA and p<0.01 by Bonferroni's multiple comparison test between Tg PrP+ and PrP–, and WT mice for n comprised between 5 and 14).
(TIF)

Figure S2 **Normal development of germinal centers and PrPc expression in secondary lymphoid organs of Tg mice.** (A) H&E staining of paraffin sections of spleens from WT and Tg mice on a PrP+ and PrP– background. Germinal centers in spleens of Tg mice have a normal size and architecture (10X). (B) Immunohistochemical staining of PrPc on frozen sections of spleens from WT and Tg mice (40X).
(TIF)

Figure S3 **PrPc expression is normal in PrP+ Tg mice.** (A) Immunohistochemical staining of PrPc was performed on frozen sections of cerebellum as described in Materials and Methods. PrPc accumulates in the white matter (10X). (B) Anti-PrPc labeling was performed on total brain cells mechanically dispersed in the presence of DNAase. Cell suspensions were incubated with a FITC-conjugated SAF61 Ab at 10 µg/ml. The overlay represents respectively brain cells of a WT PrP+ mouse (green line), a PrP+ Tg mouse (dark pink line) and a PrP– Tg mouse (dashed blue line).
(TIF)

Figure S4 **Higher frequency of IFN-γ secretors among CD4^+^ T cells from** **PrP–** **Tg versus WT mice.** T cells were collected from mice primed with peptide PrP_158-187_ 10 days earlier and subsequently incubated in vitro for 24h as described in Materials and Methods. Each number on the horizontal axes corresponds to an individual mouse. Error bars show standard error of average number of spots in at least 3 wells. The experiment was repeated 3 times.
(TIF)

Figure S5 **Significant increase in the percentage of CD4^+^ BV12^+^ T cells in lymph nodes after priming with peptide PrP_158–187_.** Cells were stained as described in Materials and Methods and analyzed by FACS. Data represent the compilation of 3 experiments. Statistical analysis was performed using one way variance analysis and Bonferroni's multiple comparison tests.
(TIF)

Figure S6 **An overview of TRAV family usage by BV12^+^ CD4^+^ T cells from Tg primed or naive mice on a PrP+ or** PrP– **background.** (A) TRAV profiles of naive BV12^+^ T cells from PrP– mice. (B) TRAV profiles of naive BV12^+^ T cells from PrP+ mice. (C) TRAV profiles of primed BV12^+^ T cells from PrP– mice. (D) TRAV profiles of primed BV12^+^ T cells from PrP+ mice.
(TIF)

Figure S7 **PrPSc content at terminal stage.** (A) Western blots were performed as described in Materials and Methods on brains of mice culled at their respective terminal stage. Mice #1 and #2 belonged to the group transferred with BV12-negative T cells. Mouse #3 was transferred with BV12^+^ T cells with no boost and mice #4 and #5 received BV12^+^ T cells further boosted. (B) Absence of detectable PrPSc in the spleen and the brain of the infected mouse which had received BV12^+^ T cells plus boosts and was still free of symptoms at 350 dpi. The tissues culled at terminal stage of a non-treated control mouse and processed in parallel serve as a positive control.
(TIF)

Figure S8 **Expansion into CD3ε^o/o^ recipient mice of transferred BV12^+^ CD4^+^ T cells.** Blood samples were collected at 90 dpi. Each quadrant represents an individual mouse. Percentages of BV12^+^ T cells in quadrants are relative to total number CD4^+^ T lymphocytes.
(TIF)
