# Supplementary figures and images for: Th2-polarised PrP-specific Transgenic T-cells Confer Partial Protection against Murine Scrapie
Source: PLoS Pathog. 2011 Sep 1;7(9):e1002216. doi: 10.1371/journal.ppat.1002216 (PMC3164648; doi:10.1371/journal.ppat.1002216)

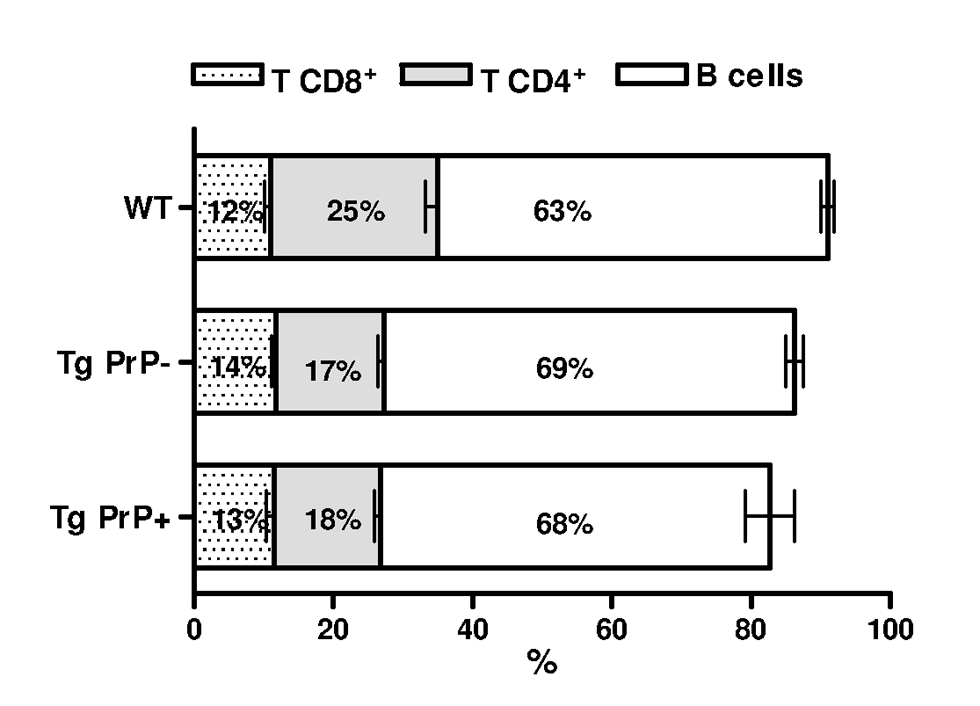

Supplement: Figure S1 — Percentages of CD4+, CD8+, and B cells in Tg mice. Percentages were measured by flow cytometry. CD4+ T cell proportion in Tg mice is slightly but significantly reduced (p<0.001 by ANOVA and p<0.01 by Bonferroni's multiple comparison test between Tg PrP+ and PrP–, and WT mice for n comprised between 5 and 14). (TIF) [file ppat.1002216.s001.tif]

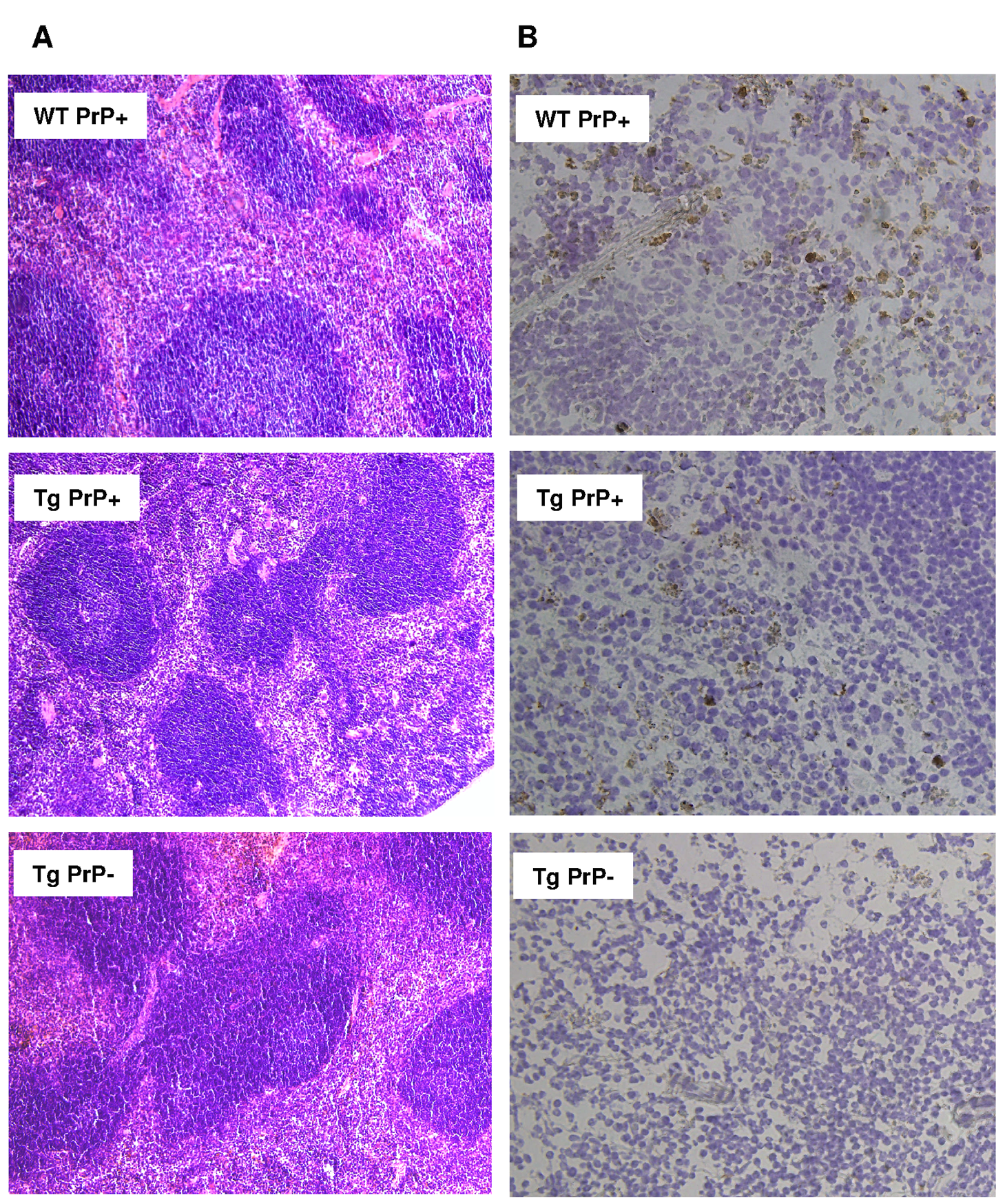

Supplement: Figure S2 — Normal development of germinal centers and PrPc expression in secondary lymphoid organs of Tg mice. (A) H&E staining of paraffin sections of spleens from WT and Tg mice on a PrP+ and PrP– background. Germinal centers in spleens of Tg mice have a normal size and architecture (10X). (B) Immunohistochemical staining of PrPc on frozen sections of spleens from WT and Tg mice (40X). (TIF) [file ppat.1002216.s002.tif]

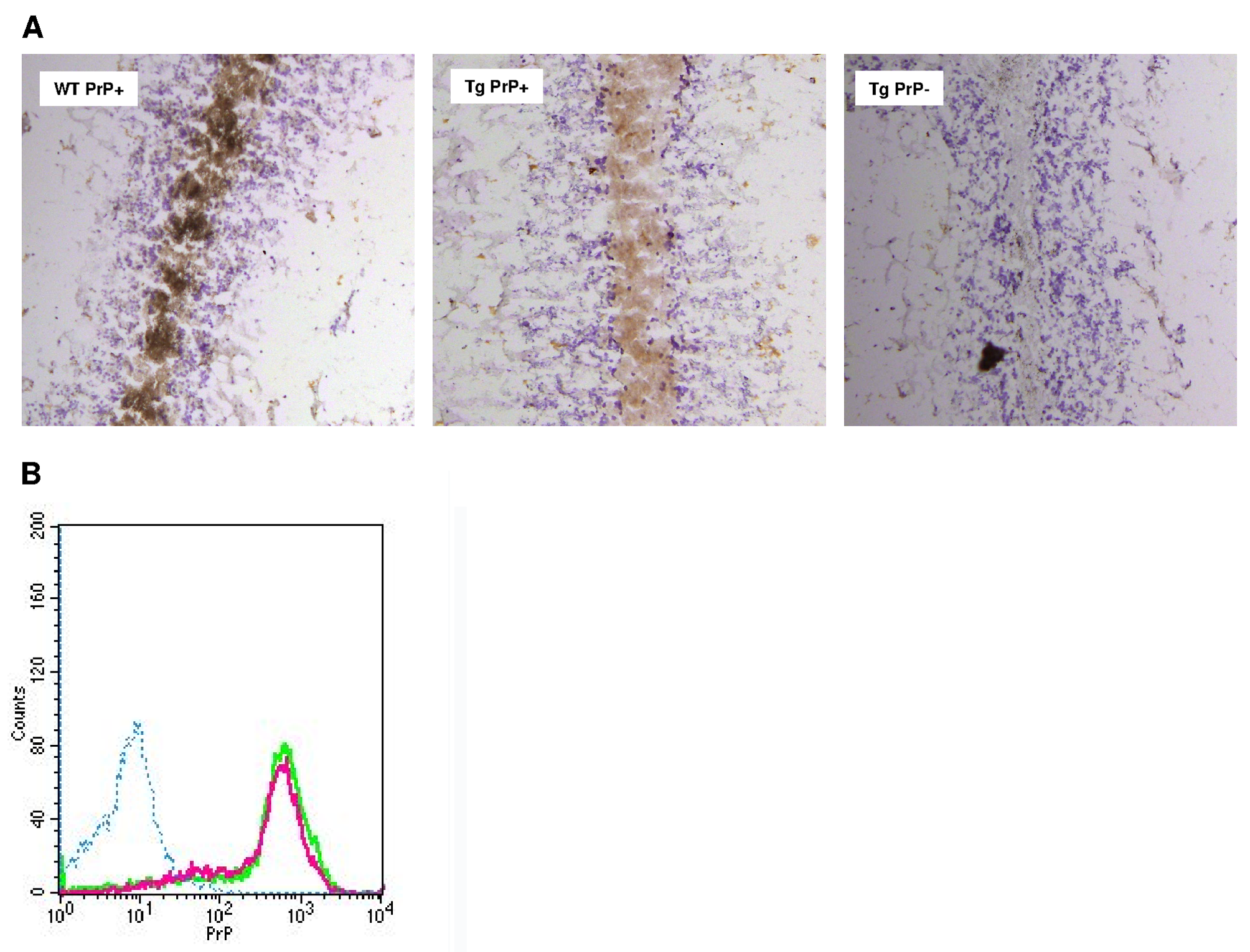

Supplement: Figure S3 — PrPc expression is normal in PrP+ Tg mice. (A) Immunohistochemical staining of PrPc was performed on frozen sections of cerebellum as described in Materials and Methods. PrPc accumulates in the white matter (10X). (B) Anti-PrPc labeling was performed on total brain cells mechanically dispersed in the presence of DNAase. Cell suspensions were incubated with a FITC-conjugated SAF61 Ab at 10 µg/ml. The overlay represents respectively brain cells of a WT PrP+ mouse (green line), a PrP+ Tg mouse (dark pink line) and a PrP– Tg mouse (dashed blue line). (TIF) [file ppat.1002216.s003.tif]

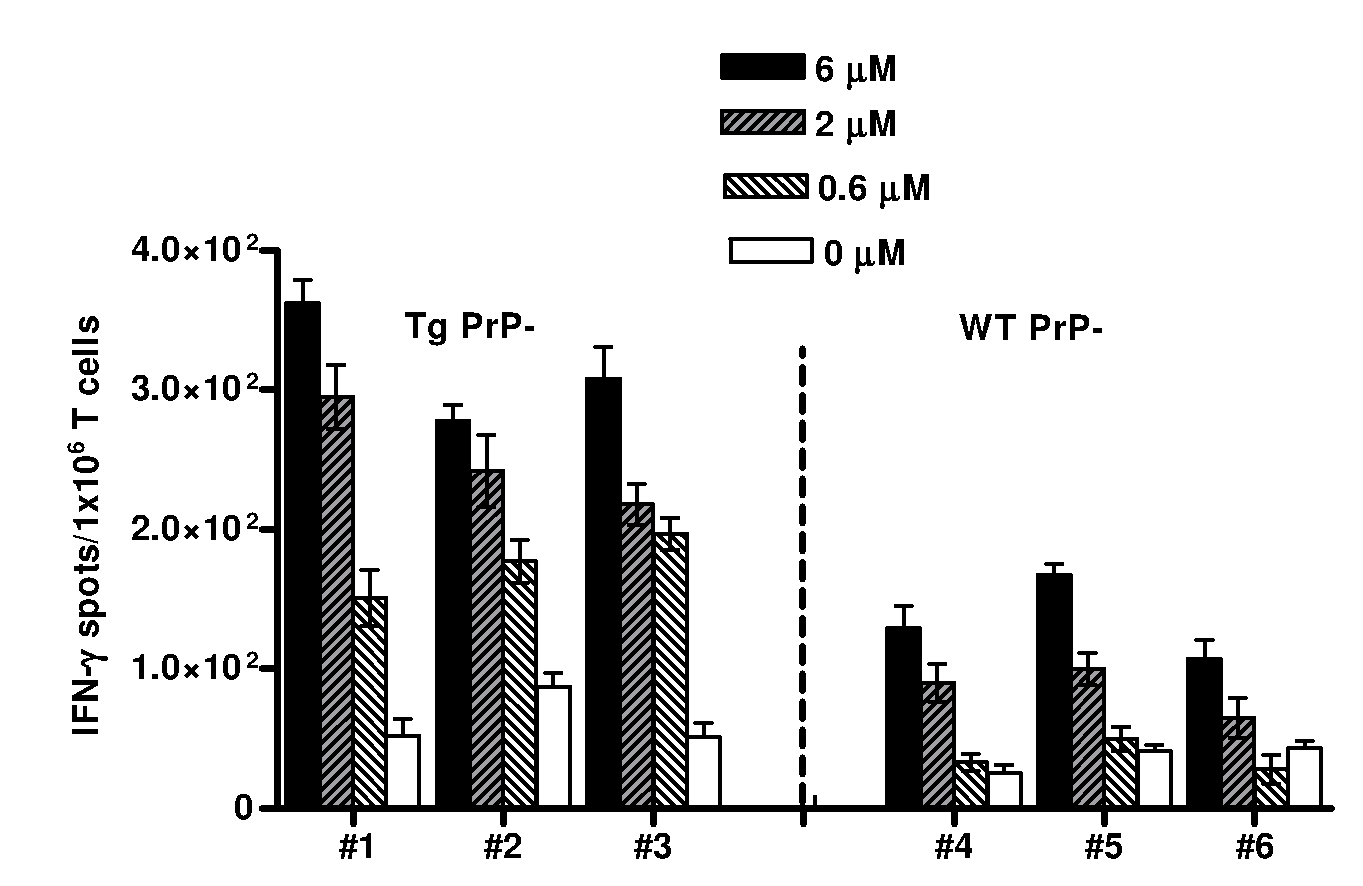

Supplement: Figure S4 — Higher frequency of IFN-γ secretors among CD4+ T cells from PrP– Tg versus WT mice. T cells were collected from mice primed with peptide PrP158-187 10 days earlier and subsequently incubated in vitro for 24h as described in Materials and Methods. Each number on the horizontal axes corresponds to an individual mouse. Error bars show standard error of average number of spots in at least 3 wells. The experiment was repeated 3 times. (TIF) [file ppat.1002216.s004.tif]

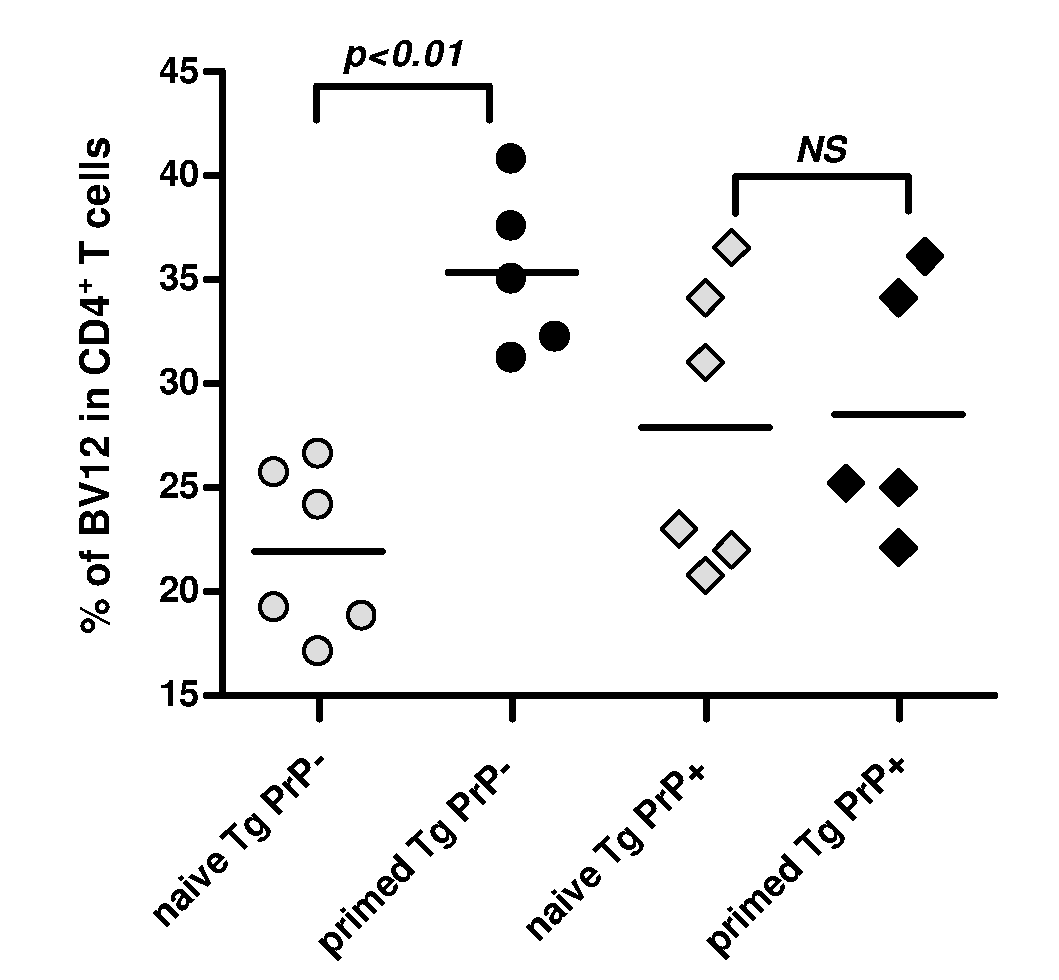

Supplement: Figure S5 — Significant increase in the percentage of CD4+ BV12+ T cells in lymph nodes after priming with peptide PrP158–187. Cells were stained as described in Materials and Methods and analyzed by FACS. Data represent the compilation of 3 experiments. Statistical analysis was performed using one way variance analysis and Bonferroni's multiple comparison tests. (TIF) [file ppat.1002216.s005.tif]

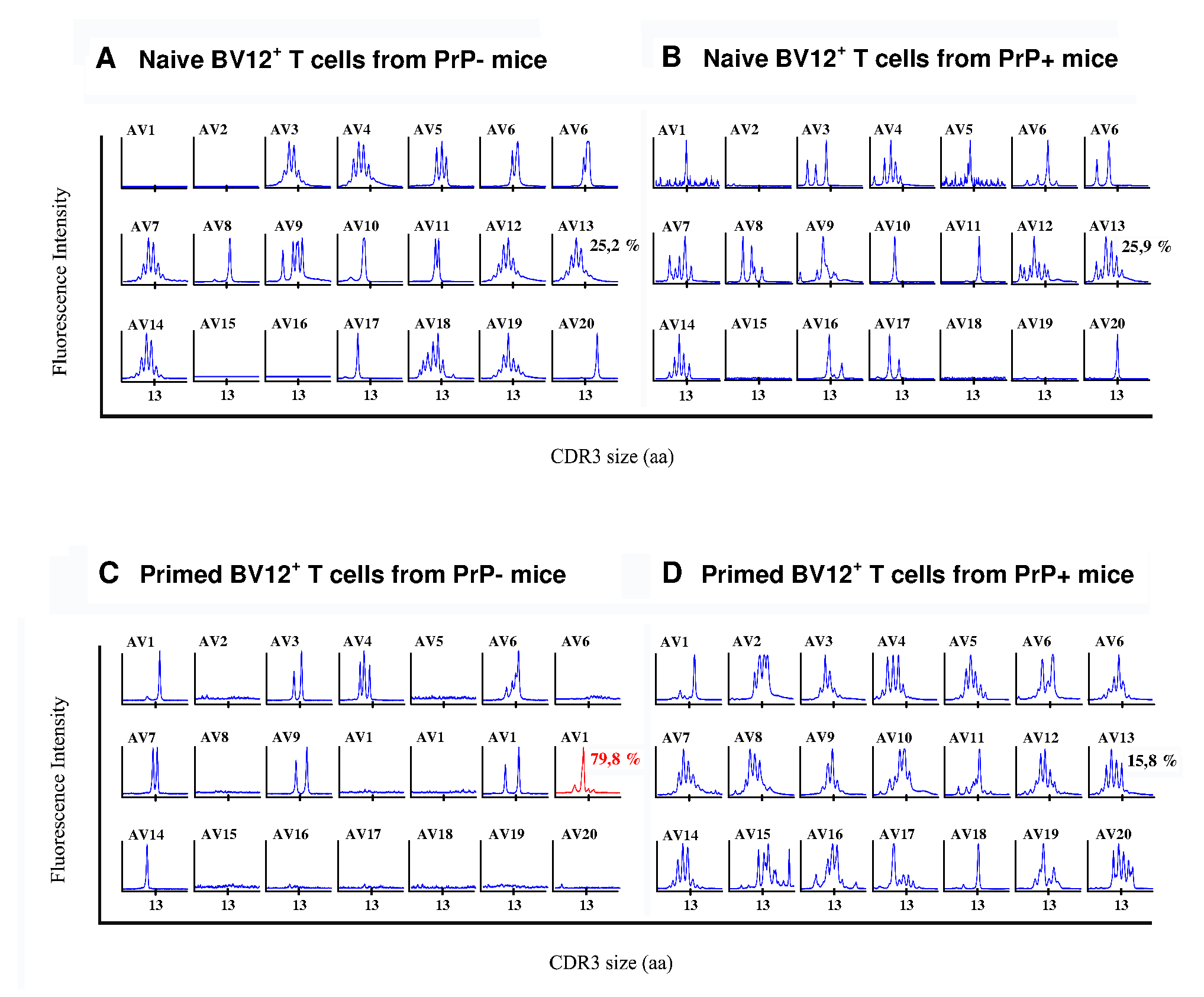

Supplement: Figure S6 — An overview of TRAV family usage by BV12+ CD4+ T cells from Tg primed or naive mice on a PrP+ or PrP– background. (A) TRAV profiles of naive BV12+ T cells from PrP– mice. (B) TRAV profiles of naive BV12+ T cells from PrP+ mice. (C) TRAV profiles of primed BV12+ T cells from PrP– mice. (D) TRAV profiles of primed BV12+ T cells from PrP+ mice. (TIF) [file ppat.1002216.s006.tif]

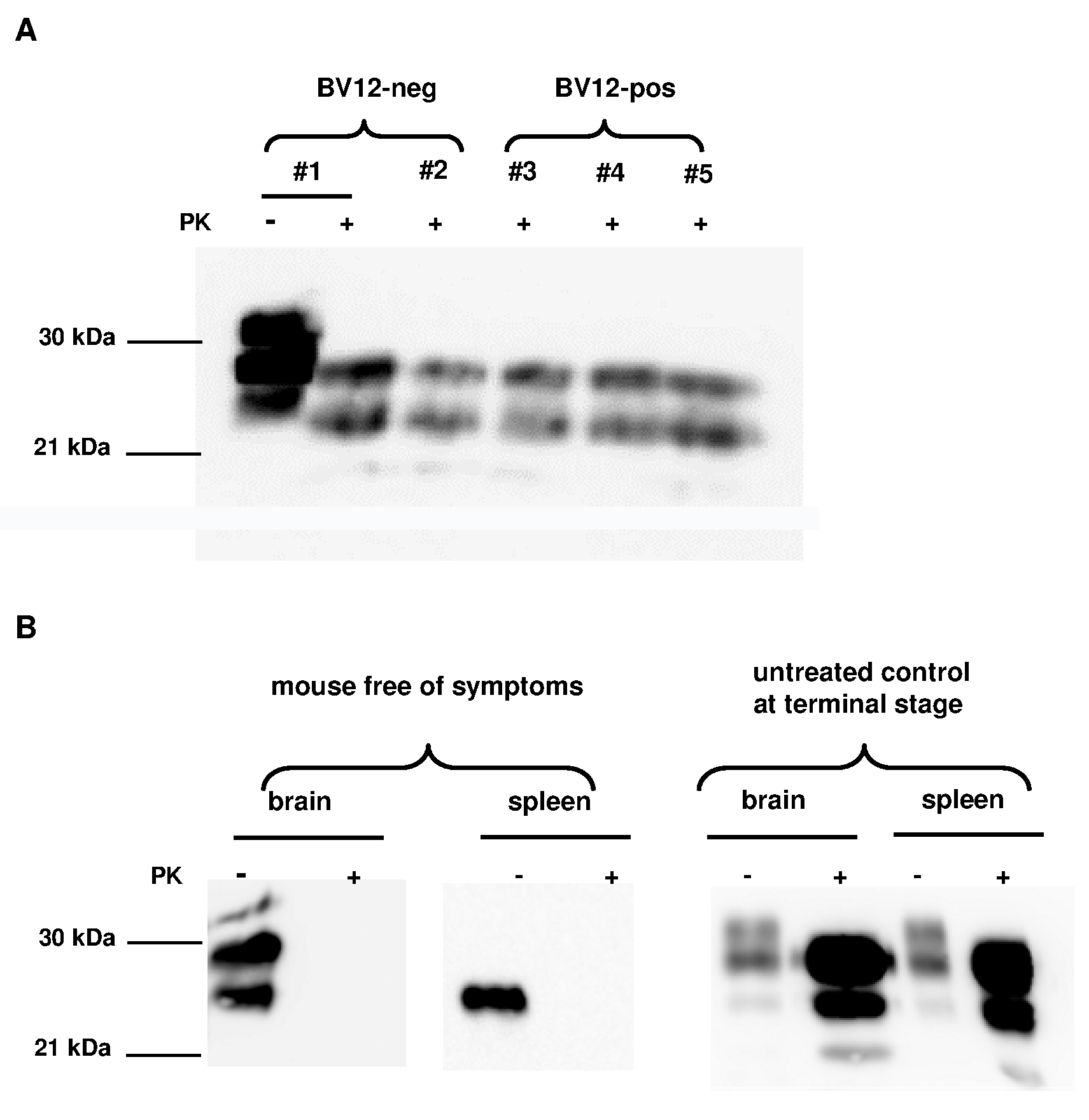

Supplement: Figure S7 — PrPSc content at terminal stage. (A) Western blots were performed as described in Materials and Methods on brains of mice culled at their respective terminal stage. Mice #1 and #2 belonged to the group transferred with BV12-negative T cells. Mouse #3 was transferred with BV12+ T cells with no boost and mice #4 and #5 received BV12+ T cells further boosted. (B) Absence of detectable PrPSc in the spleen and the brain of the infected mouse which had received BV12+ T cells plus boosts and was still free of symptoms at 350 dpi. The tissues culled at terminal stage of a non-treated control mouse and processed in parallel serve as a positive control. (TIF) [file ppat.1002216.s007.tif]

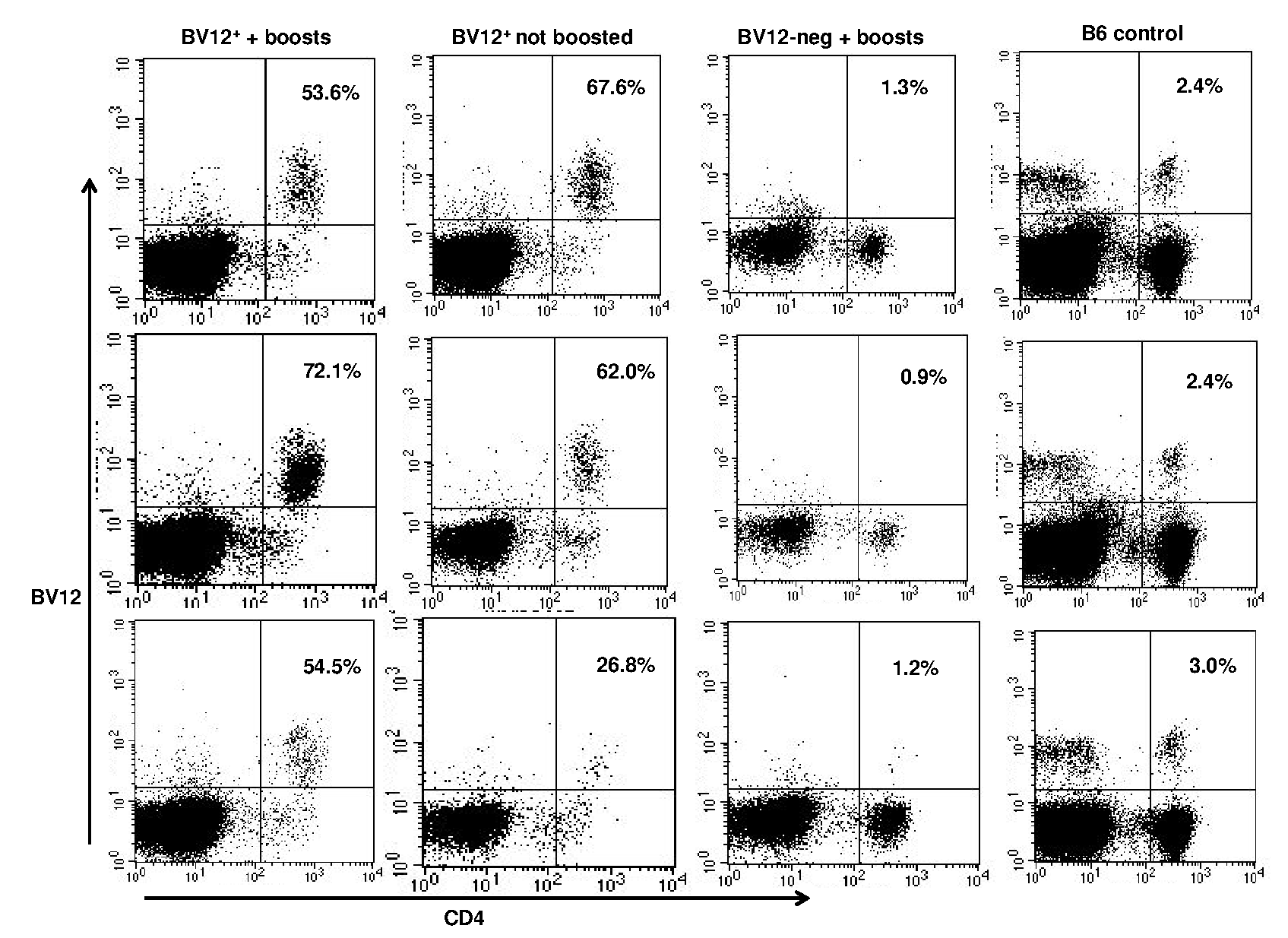

Supplement: Figure S8 — Expansion into CD3εo/o recipient mice of transferred BV12+ CD4+ T cells. Blood samples were collected at 90 dpi. Each quadrant represents an individual mouse. Percentages of BV12+ T cells in quadrants are relative to total number CD4+ T lymphocytes. (TIF) [file ppat.1002216.s008.tif]
